# A model curriculum in sexual medicine for undergraduate education in Europe

**DOI:** 10.12688/openreseurope.16146.1

**Published:** 2023-09-19

**Authors:** Carlo Matteo Di Dionisio, Johannes Bitzer, Marianne Greil-Soyka

**Affiliations:** 1 Endocrinology and Medical Sexology (ENDOSEX), Department of Systems Medicine, Universita degli Studi di Roma Tor Vergata, Rome, Lazio, Italy; 2 University Hospital - Department of Obstetrics and Gynecology, Universitat Basel, Basel, Basel-Stadt, Switzerland; 3 Austrian Academy for Sexual Medicine (OEASM), Salzburg, Austria, Austria

**Keywords:** medical education, undergraduate curriculum, sexual health, sexual medicine

## Abstract

Sexual health has been recognized as an essential component of overall health and wellbeing. The current article aims, first, to review the current state of sexual health education in undergraduate medical curricula, identifying gaps, needs and challenges.

The main part of this paper describes the development and content of an undergraduate sexual medicine curriculum based on a clear concept of the competencies, students should learn regarding knowledge, skills and attitudes.

The content is based on a biopsychosocial understanding of human sexuality elaborated by international experts from different European countries integrating basic knowledge in biology, psychology, sociocultural and political sciences, preventive medicine, and the various therapeutic approaches to help women, men and couples with sexual health problems on a primary care level. In order to enable students to learn the basic skills of sexual history taking and basic sexual counselling two educational videos were produced.

The material presented is part of the European Cooperation in Science and Technology (COST) supported project European Sexual Medicine Network (ESMN). The material provided can serve universities to give the training as a 25–30 hours course equivalent to 1 ECTS.

## Introduction

The present article aims to describe the process that led to the development of a novel sexual medicine curriculum for undergraduate students. This curriculum was created throughout 4 years by a working group of international experts under the network association nominated European Sexual Medicine Network (ESMN) ^1^, which is an European Cooperation in Science and Technology (COST) Action (CA18124).

The working group had the following tasks

1) Assess gaps and inequalities regarding undergraduate university education in sexual health, sexual health care and sexual medicine in Europe
2) Analyse the difficulties and challenges regarding the creation of a broadly applicable and suitable program for faculties in different countries
3) Define a competency- based curriculum with learning objectives regarding knowledge, skills and attitudes based on the biopsychosocial model of sexuality and sexual health
4) Elaborate and provide the educational material for use in teaching programs integrated into the existing medical and psychology curriculum

As discussed in section 3 and 4, the rationale at the foundation of this effort lies in the current lack of effective and inclusive sexual health education in undergraduate, first level educational curricula of European universities. The discussion builds up on the current fragmented state of education and the resulting lack of expertise for healthcare professionals in addressing sexual health issues with patients. It follows with an overview of the challenges that may hinder the development and implementation of a comprehensive sexual medicine curriculum. 

To fill these gaps and harmonize basic university training in sexual health and sexual medicine, the COST Action European Sexual Medicine Network established a working group to elaborate a model curriculum in sexual medicine for undergraduate education in Europe. This working group consisted of experts representing different disciplines and fields involved in sexual medicine and sexual health care and different regions in Europe and beyond (see Acknowledgments). 

 The results of this shared effort are described in the main part of the final section of this article.

## Methods

For task 1 and 2 an extensive search of all materials related to the topic was carried out in the PubMed and Google Scholar search engines. Relevant research articles focusing on sexual health education in undergraduate medical education published in the period 2006–2023 were included in the review. Relevant articles were selected based upon relevance with the current review objectives and analyzed. Keywords used in the search included sexual health, medical education and curriculum (namely sexual health AND medical education; sexual health AND undergraduate medical education; sexual health AND curriculum). The articles published only in the English language were included for the current review. The collected information is presented in the following two sections.

For tasks 3 and 4 the working group consisting of sexual medicine experts in the field of gynecology, urology, social science, public health, preventive medicine, internal medicine and endocrinology from different European countries had in person and virtual meetings which followed a modified Delphi procedure to achieve consensus ^2^.

For all steps described below the chair and vice chair summarized discussions and inputs coming from the experts, sent the material back to the group members to get feedback and corrections which were then integrated and sent back to the group members for evaluation and feedback. Where necessary this procedure was repeated.

In a first step the group had to define the competencies undergraduates should obtain and the related learning objectives. In the next step the group members were invited to suggest topics to be covered by the program which were considered to be essential parts of basic knowledge. After having agreed on the topics experts were invited to contribute to the topics chosen by sending slides to the chair and vice chair. Each slide had to be accompanied by learning objectives, text, references and self-assessment questions for the students. The chair and vice chair integrated the material into a first format of the 10 topics and sent the proposals out for review and comments from the group. This procedure was repeated several times to arrive at the final format which is shown in the text below and accessible through the internet link given at the end

## Results

### Gaps and inequalities regarding education and training in sexual health care across Europe

Sexual health is closely linked to both physical and mental wellbeing ^3– 6^. It encompasses the life of the individual as a fundamental component, constantly evolving and changing with it. Accordingly, sexuality is closely related to general health and for this reason, various medical conditions and treatments can have a negative impact on a person's sexual function, interpersonal relationships and well-being ^7, 8^. The importance to not overlook sexuality appears evident at the time of a cancer treatment, for example, where most often the side effects impact heavily on the interpersonal (intimate) relationship of the patient and may affect therapy adherence ^9^; or, in those cases in which a sexual dysfunction can represent a marker of an underlying, non-diagnosed, illness ^10, 11^. Therefore, it is crucial for healthcare providers to consider and address their patients' sexual health alongside other medical issues.

Various barriers have been identified at the time of a clinical visit, that prevent both healthcare providers and patients to talk freely about sexual health issues. Among these, some of the most frequently reported are: lack of training in communication, fear of offending the patient, discomfort with discussing sexual health, lack of education and competence in treating sexual health issues and lack of time in the appointment ^12^. Patients view sexual health as an important aspect of their well-being and happiness, but they are afraid and may feel ashamed to address intimate issues. They expect their physicians to first open the discussion about sexual health problems ^13– 16^. However, many doctors feel they lack adequate education on sexual health and, consequently, do not routinely inquire about sexuality because they feel incompetent or are afraid to lose too much time ^17– 19^.

A significant contributing factor to this, identified repeatedly in the literature, is the insufficient attention given to sexual health education in medical school ^5, 20, 21^.

Despite the broadly acknowledged importance of sexual health education in medicine, many medical schools still do not offer sufficient training on this topic ^19, 22, 23^. This gap in education must be addressed to better equip future physicians with the knowledge, skills and attitudes necessary to provide comprehensive care for their patients ^24^. Medical schools should prioritize the inclusion of sexual health education in their curriculums, providing students with the resources and opportunities to develop their competence in this area. This matters also for those universities that may already implement some components of sexual medicine but do so only for topics related to sexually transmitted diseases, pregnancy, reproductive anatomy, and infertility. A comprehensive sexual medicine education should be composed of a multidisciplinary curriculum, providing a health-oriented, biopsychosocial view of human sexuality ^25, 26^.

Multiple surveys have identified this lack in a variety of countries, especially in the USA, Brazil and Northern Europe ^5, 12, 27, 28^. The European region, in particular demonstrates a lack of standardized and unified sexual education inside the existing training curricula ^29, 30^. Results from a survey conducted by the European Federation of Sexology (EFS) ^31^ on existing programs and certifications on sexology in Europe, identified 6 different training models in 25 countries:

- Medical model (in France, Czech Republic, Russia, Poland, Romania and Latvia);
- Clinical model, integrating medical and psychological approaches (in Italy, Netherlands and Turkey);
- Distinct training in clinical sexology and human sexuality (in Germany, Austria and Spain);
- Sex therapy model (in Portugal, Austria, Greece, Israel, Croatia, and United Kingdom);
- Human sexuality model (in Belgium and Switzerland);
- Nordic human sexuality model (in Sweden, Denmark, Finland, Norway, Iceland and Estonia);

The available education in the field of sexology typically consists of national programs that encompass a range of courses. These programs may lead to a degree, certificate, or diploma in different areas and levels of sexology *(trained in; doctor in; sexologist; sex therapist; etc.).* The majority of this training focuses on providing additional education to professionals who have already acquired a primary educational level. For this reason, it is more common to find sexual health and medicine education in complementary, master or postgraduate courses. 

In the last two decades the postgraduate education in sexual health, sexual medicine and sexology has seen an enormous growth, mainly through European and International Scientific societies. In particular: International Society for Sexual Medicine (ISSM), European Society for Sexual Medicine (ESSM), International society for the Study of Women’s Sexual Health (ISSWSH) and several national societies as well as various universities. The concern remains that sexuality and sexual health care are not well integrated into the undergraduate education for future health care professionals including training in medicine and psychology, as at best sexuality education may be offered as elective courses within the basic health professional curriculum. In most European universities, sexology is not an essential component of the teaching curriculum, even for medical students. This marginalization within the existing educational system makes it difficult to enable future health care professionals to provide basic sexual health care.

### The challenge(s) to create a training program in basic sexual health care for undergraduates

Developing a comprehensive sexual medicine curriculum does not concern only the ability to resume and integrate a variety of multidisciplinary concepts around sexual health. The number of challenges that must be considered is not insignificant as they encompass the whole educational environment of sexual medicine.

***Scientific evidence and biopsychosocial, multidisciplinary approach***

The first challenge is to create a curriculum that respects quality criteria, the most important one being that the information provided is **scientifically accurate**, **complete, up-to-date and adapted to its final receivers**. It should not be limited to anatomy and pathology, and should instead provide knowledge about aspects of biology, psychology, and social factors altogether (i.e., the biopsychosocial model of human sexuality) ^32^. It should not overlap with the same depth topics that are already taught in medical courses, but should identify them accordingly, so that students can learn and integrate available knowledge inside a more holistic approach. The development of such an educational package should be guided by inputs from different healthcare specialties and disciplines: obstetrics, gynecology, urology, psychiatry; sex therapists, psychologists, and sexologists; and public health experts ^24, 33^. Another step towards this holistic approach would aim to create a working group composed of international experts from different countries and regions, to assure the inclusion of different sociocultural and political backgrounds important to understand patients’ concerns and sexual problems.

From a didactic point of view the challenge lies in harmonizing topics of human biology, psychology and social and political aspects, without overweighting too much a single topic. The educational level should be comprehensive, but not go beyond the level of the current courses on human anatomy, pathology, etc. It should result in a balanced biopsychosocial educational package on sexual health without deepening or discounting too much. Most importantly, the sexual medicine curriculum should include topics that are missing or not covered enough from the current available courses (i.e. gender minorities and inclusive care, digital sexuality, non-normative sexual behavior, sexuality and aging, sexual coercion and violence, sexual rights, to cite a few) ^24^, and should be aimed at developing those skills that practicing physicians identify as challenging (i.e. sexual history taking) ^26^.

Another challenging aspect is that this curriculum should, where possible, be applied to a larger pool of students other than just those of medical schools. The rationale is simple: given the strict linkage between health and sexuality, it is highly likely that healthcare professionals of all kinds will come across patients who have questions or difficulties related to their sexual health ^24^. Moreover, it is strongly suggested that this should start during the first years of University, rather than the latest and or during residencies, as difficulty in discussing the sexual health of patients emerges prior to graduation ^34^ and is easier to overcome if students can practice during their routine care ^28^.

***Delivery and implementation of the curriculum***

Effective delivery of the Curriculum to students faces three main challenges. 

The first consists of tailoring the program to the social climate of the students. Current generations, studying today at university level, were born and raised through a digital era, to which their ability to learn is connected ^25^. In this sense, implementation through internet platforms, modular and accessible anytime, anywhere to learn and practice at their will could represent a dissemination modality to be preferred, especially considering the evolution of digital technologies for self-help and sexual health ^35^.

The second, far more important, is that this tailored and timed education should connect knowledge acquisition with attitude formation and skills practice. To detach from a purely theoretical approach and instead opt for an experiential learning approach: through the use of roleplay, workshops, case discussion and standardized patients; through (multidisciplinary) team-work and direct constructive feedback from peers and educators. The resulting increase in knowledge and *savoir-faire* allows one to develop a professional attitude, as discussed in the next section.

The third challenging aspect relates to both content creation and student education. A key component of the curriculum is to develop and foster a professional stance. By teaching about the diversity of sexual expression, the curriculum shall prepare to meet patients with different sexual values and expression in respect to one’s own. There is no interest in altering the student’s subjective view of sexuality, but rather the objective is to raise awareness and the ability to maintain an objective, unbiased, non-judgmental evidence-based stance during patient care for a sexual concern ^5, 26^.

Finally, the last challenges regard the mode of implementation, the University structure (Faculty, Educational board) and the sustainability of the curriculum.

An implementation modality to bring sexual health care in medical schools would be to integrate its components into multiple courses throughout the years of training ^5, 26, 36^. Considering the current limited space of medical schools curricula for new courses, and the general burden placed on students, the above mentioned proposal is not feasible. A solution to this is represented by shorter implementations, such as modules as short as two days of activities, that connect evidence-based knowledge and skills practice. In the literature, this kind of implementation has been preferred by students, implemented by universities ^37^, and has demonstrated positive lasting effects even after ten years ^20^.

 Another limitation is represented by a lack of resources, training and time by the faculty of the interested medical school. In this case, external experts could be invited to provide education on sexual health topics, both to students and to willing faculty members ^25^. At the same time, formal approval by the university educational board might become another complexity.

 A final challenge concerns the educational curriculum maintenance and follow up. A comprehensive sexual medicine curriculum, as stated, would need to be updated according to new relevant research, alongside the faculty of experts that may present it; it would need an administrative body, to regulate and manage financial and human resources, and a disseminative body, to bring the curriculum to the attention of willing stakeholders and institutions. 

To conclude, both students, specialists, researchers and experts have highlighted the need for further sexual medicine education within the undergraduate years. For this reason, a well-timed and implemented curriculum might allow for a seeding process for sexual healthcare, as even a short training program (two days or less) can have a long-lasting impact on the medical competence and confidence in addressing sexual health issues, all to the benefit of patients. 

### The design and the content of the curriculum for Sexual Medicine for undergraduates agreed upon by the international working group

Working group three designed the curriculum based on the concept of a competence-based program integrating knowledge, skills and attitudes ^38^.

***Competencies and learning objectives of the curriculum***

Having participated in the course undergraduate student should be able to successfully:

- Perform a sexual history including assessment of sexual health risks and sexual concerns, encourage questions and create an open, confidential and non-judgmental atmosphere following the principles of patient centered communication and patient/professional relationship based on trust and respect
- Counsel patients on sexual health protection and promotion (contraception, STIs, violence)
- Encourage questions and inform and educate patients and their partners about the basic facts of the anatomy, physiology and psychology of the human sexual response in women and men and their partners
- Address proactively sexual wellbeing and sexual function in a respectful non-invasive manner
- Take care of patients with sexual problems and concerns in general as well as in the context of disease
  - By practicing patient or couple centered counseling based on proactive respectful asking, empathic listening and providing evidence-based information with respect to the problem presented
  - By informing patients (couples) about therapeutic options and provide referral if desired by the patient (couple)
  - Communicate, educate and counsel non only the individual patient but have the skills to include the partner in the process (dyadic setting)

***Knowledge***

Following a thorough analysis, the working group concluded that the curriculum should include ten major topics covering the necessary background knowledge undergraduate student should have with respect to sexual health and basic sexual health care:

Topic 1: Sexual health and sexual rights

- Sexual health concepts, definitions
- What is sexual health? Importance of sexual health for general health
- Dimensions of sexuality (reproduction, bonding, pleasure, etc); Variety of sexual expression

Topic 2: The sexual body. Biology of human sexuality

- From genes to bodies
- Biological (Anatomical and Physiological) processes and constituents of human sexuality
- Development, Human Response, Anatomy, Physiology, Endocrinology, Biological systems approach

Topic 3: Psychology and Sexuality

- Psychological perspectives and theories
- Psychotherapeutic methods to help women and men with sexual problems
- Barriers and Facilitators to lead a self-determined sexual life

Topic 4: Threats to sexual health, sexuality related health risks; prevention and public health aspects

- Unintended pregnancies and unsafe abortion;
- STIs inc. HIV
- Sexual violence
- Prevention, maintenance and promotion of sexual health

Topic 5: Female sexual dysfunction

- Definitions and Prevalence
- Diagnosis (biopsychosocial)
- Types of treatment (biopsychosocial)

Topic 6: Male sexual dysfunction

- Definitions and Prevalence
- Diagnosis (biopsychosocial)
- Types of treatment
- Sexual Dysfunction and the couple

Topic 7: Medical conditions and sexuality

- Medical illnesses and treatment impact on sexuality
- Medical conditions (including psychiatric morbidity) and sexuality and the biopsychosocial approach in medical sexology

Topic 8: Sexual minorities

- Sexual health of minorities
- LGBTQI*, Sexual Needs
- Need based care

Topic 9: Digitalization

- Websites, Apps, Serious Games
- Sexual Health Promotion
- Sexual Diversity

Topic 10: Compulsive sexual behavior, Paraphilias

- Definitions
- Contributing factors and risks
- Legal framework
- Therapeutic approaches

This knowledge should be transmitted to students through a series of lectures with learning objectives, slides, text with references and questions

In a shortened integrated version of the knowledge part of the curriculum the content should be summarized along 5 modules:

- Module 1: Sexuality, Biology, Psychology, Sexual Medicine
- Module 2: Threats to Sexual Health, Sexual Dysfunctions
- Module 3: Sexuality during the life course, Diseases and sexuality
- Module 4: Diversity, Sexual minorities, Digitalization
- Module 5: Compulsive Sexual Behavior, Paraphilias

***Skills***

The skills training part of the curriculum should focus on the specific challenges regarding communication and counseling on sexual and reproductive health. It is important to address sexual health issues in a patient centered way and when addressing issues in a couple to respectfully consider both partners perspectives.

Patient centered communication in Sexual Medicine can include a whole range of topics such as reassuring patients of privacy and explaining confidentially, making efforts to ensure trust and openness, and avoiding assumptions (on topics like sexual orientation, gender identity, monogamy, sexual activities, or age-related practices).

This part should be taught by videos and discussions with students (i.e. role play), in particular:

- Video 1: Sexual history taking based on the concept of the variety of sexual expression
- Video 2: How to help patients presenting a sexual problem in the consultation

***Attitudes***

Undergraduate students should be made aware of their beliefs and values regarding sexuality and the impact of these on their care for patients. It will also be important for students to understand the large variability of sexual expressions and develop an open non-judgmental attitude.

### The final version of the curriculum

**After having elaborated and agreed on the main components of a competence-based program in sexual medicine for undergraduates the working group developed the material to allow universities to implement the teaching of basic sexual medicine and sexual health care in existing undergraduate curricula.**

The material can be used according to the needs of the undergraduate educational program of medical schools (universities) and academic institutions for the teaching of psychology defined by the respective responsible academic board or dean. Singular topics and a combination of topics can be integrated into the curriculum in the Bachelor or Master program (divided or total). In universities not following the Bologna system the material can be used according to the respective educational needs.

***Teaching knowledge***

**Figure 1.**
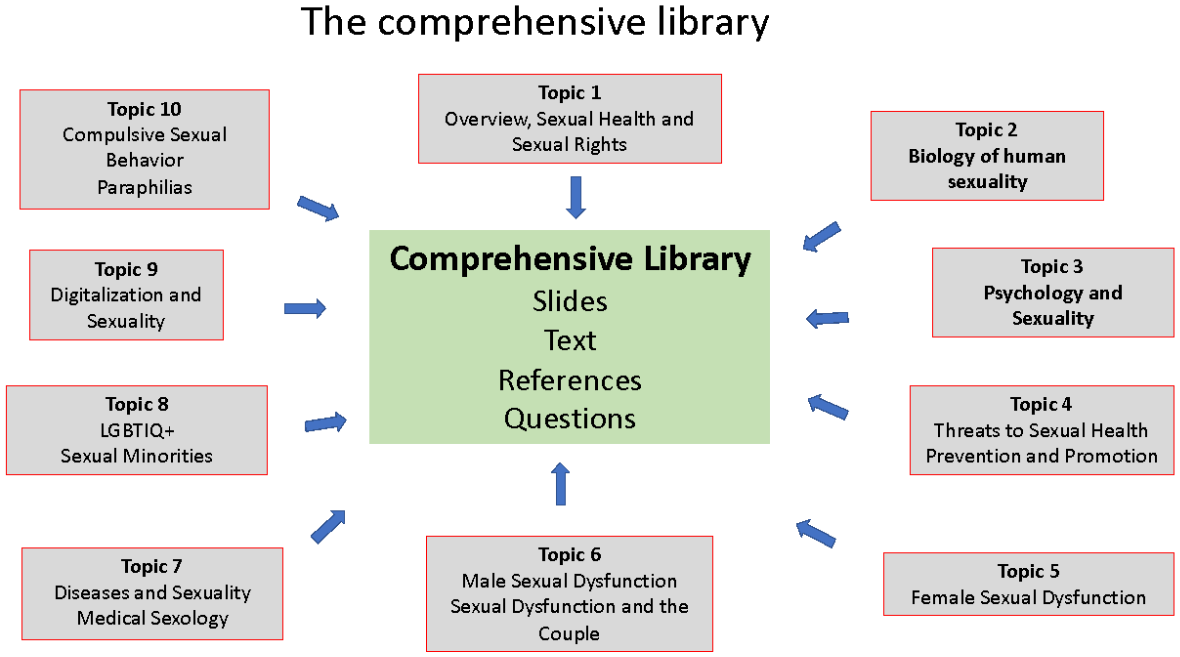
The comprehensive library.

This part of the curriculum is developed based on the broad understanding of human sexuality as an experience and behavior which integrates biological and psychosocial factors and processes interacting with each other. Based on this concept the 10 topics agreed upon are covered by a respective slide set. Each slide has defined learning objectives, text, references and questions for self-control. These 10 topics represent the comprehensive library (see Figure 1).

**Figure 2.**
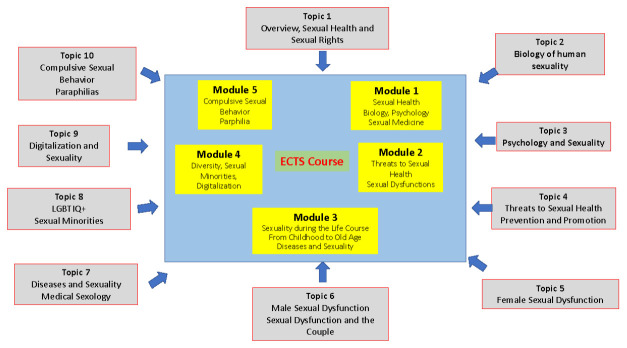
Modules Example.

These topics provide the core body of information which can be used by lecturers, trainers and teachers according to their needs and their special interests. For teaching purposes this core body of information is structured along five modules (see Figure 2).

### Module 1: Overview about definitions of sexual health; basic biology and psychology of human sexuality

Module 1 provides basic knowledge about definitions of sexual health with the implications for public health programs and multidisciplinary sexual health care for the individual provided by different health care professionals whose basic training is built on understanding the main principles and elements of the biology and psychology of the human sexual response.

The slides, text, references are made to help students

- Understand human sexuality as an integral part of human existence and behavior and know and understand the concepts of sexual health and sexual rights
- Know about the determinants and contributing factors of sexual health on the international, national and individual level and understand the linkage between sexual health promotion and reproductive health
- Know about basic models of understanding the human sexual response
- Understand the biology of the sexual response from a systemic perspective with the different interactive elements linked to each other with top down and bottom up signaling pathways acting together in accordance with dual control model (exciting, neutral, inhibiting) including the response of the peripheral sexual organs
- Understand and know about the different psychological perspectives and concepts regarding human sexuality from bioevolution to cognitive behavioral, psychodynamic and systemic approaches
- Understand the psychological factors (motivations, thoughts, emotions, behavior, communication) contributing to a self-determined and enjoyable sexuality and the psychological factors hindering and inhibiting the development and realization of this individual sexual self-expression
- Understand the Dual Control Model of Sexual Expression from a psychological perspective
- Understand the key determinants for a pleasure-based approach to sexual health, according to the World Association of Sexology
- Understand which are the most common reasons for individuals to look for counselling and know about the wishes and needs of people regarding their sexual health
- Based on the biological and psychological factors contributing to the sexual health of the individual, understand and know about the various professionals involved in helping individuals with sexual problems., in the so- called Multidisciplinary approach

***Module 1 is a composition and shortened form of the material produced in topic 1, topic 2 and topic 3. These materials can be used by the teachers to broaden the respective chapters with text, references, questions.***

Topic 1 includes slides, text, references to help students

- Understand human sexuality as an integral part of human existence and behavior
- Know the different ways and large variety of sexual expression
- Know and understand the concepts of sexual health and sexual rights
- Know about the objectives and the definition of sexual medicine, sexology
- Know about the public health approach to sexual health
- Know about the prevalence and the variety of sexual problems
- Know about the different ways individuals can get help

Topic 2 includes slide, text, references to help students

- Understand the biology of the sexual response from a systemic perspective with the different interactive elements linked to each other with top down and bottom up signaling pathways which together act according to the dual control model (exciting, neutral, inhibiting)
- Understand the receptor and effector function of the brain with the involved brain regions
- Understand the role of hormones and neurotransmitters in the regulation of the response
- Understand the role of the autonomic nervous system

Topic 3 includes slides text and references to help students

- Understand and know about the different psychological perspectives and concepts regarding human sexuality from bioevolution to cognitive behavioral, psychodynamic and systemic approaches
- Understand the psychological factors (motivations, thoughts, emotions, behavior, communication) contributing to a self-determined and enjoyable sexuality and the psychological factors hindering and inhibiting the development and realization of this individual sexual self-expression
- Based on this the students should understand and know about the main psychological and psychotherapeutic interventions from counseling to body centered to individual and systemic psychotherapy

### Module 2: Threats to sexual health; Sexual dysfunctions

This module provides the basic knowledge about the threats to sexual health as well as the prevention and promotion of sexual health on a social and an individual level and the most frequent sexual dysfunctions in women, men and couples. The diagnostic approach to describe, differentiate and understand these dysfunctions from a biopsychosocial perspective and the various treatment options including medical and psychosocial interventions are demonstrated as overviews.

The slides, text, references are made to help students:

- Know about the major and main threats to sexual health and their impact on physical and mental health in general and sexual health more specifically
- Know about the global epidemiology of unintended pregnancies, unsafe abortion, STIs, Sexual violence, Sexual discrimination and their specific health consequences and implications on sexual health
- Know about the multilevel strategies and instruments for Sexual Health Prevention and Promotion (Laws and politics, Education, Sociocultural environment, Economy)
- Know about and the different categories of female and male sexual dysfunctions
- Know about and understand the biopsychosocial model of sexual function and dysfunction with the four dimensions
- Know and understand the different steps to establish a diagnosis in patients complaining about sexual problems: From listening and history taking to a descriptive diagnosis and from there the comprehensive explanatory diagnosis summarizing the biomedical, psychological and sociocultural factors contributing to the problem. Some of these factors are distant and predisposing, some are precipitating and some are recent and maintaining the problem
- Know about main factors contributing to female and male desire and arousal disorders
- Know about the main factors contributing to female and male orgasmic disorders
- Know about the main factors contributing to female pain disorder
- Know about the specific main factors contributing to male pain disorder
- Know and understand the multidimensional therapeutic approach to help individuals with suffering from sexual dysfunction (basic counselling, medical, psychological, psychosexual interventions, physiotherapy) with examples for Desire disorder and Erectile Dysfunction

***Module 2 is a composition and shortened form of the material produced in topic 4, topic 5 and topic 6. These materials can be used by the teachers to broaden the respective chapters with slides, text, references and questions.***

Topic 4 includes slides text and references to help students:

- Understand the macro and microfactors which endanger health sexual from the political/legal to the sociocultural to the individual level
- Know about the main threats to sexual health including unwanted pregnancies and unsafe abortions, sexually transmitted infections, sexual violence and sexual dysfunctions
- Know about the instruments to protect and maintain sexual health of the individual acting on these different levels (laws, norms, sexual education, good sexual and reproductive health care including contraception, abortion care, STI prevention)

Topic 5 includes slides, text, references and questions to help students:

- Know about the different phases of the sexual response in women and the respective dysfunctions and/or problems (Loss of desire leading to personal distress or problems in the relationship, Arousal difficulties manifesting as not getting mentally aroused and/or lack of vaginal lubrication, Difficulties to orgasm, Pain during sexual activity)
- Know how to diagnose these dysfunctions (history, examination)
- Know about the possible contributing factors (medical, psychological, interpersonal, sociocultural)
- Know about the treatment options (medical, psychological)

Topic 6 includes slides, text, references and questions to help students:

- Know about the different phases of the sexual response in women and the respective dysfunctions and/or problems (Loss of desire leading to personal distress or problems in the relationship, Arousal difficulties manifesting as not getting mentally aroused and/or lack of vaginal lubrication, Difficulties to orgasm, Pain during sexual activity)
- Know how to diagnose these dysfunctions (history, examination)
- Know about the possible contributing factors (medical, psychological, interpersonal, sociocultural)
- Know about the treatment options (medical, psychological)

### Module 3: Life course approach to sexual health, medical sexology

This module provides the basic knowledge about the development of human sexuality along the life course taking into account that sexuality is a not a fixed unchangeable dimension of life but that there are typical developmental aims and strands which expose individuals to life stage related sexual difficulties demanding age sensitive sexual health care from childhood to adolescence, midlife to the elderly individuals and couples. Diseases are part of human life and have an impact on sexual health through biological and psychosocial changes. The module provides an understanding of the different levels through which diseases and treatment can affect sexuality, how a sexual dysfunction in the context of a medical condition can be diagnosed and what the therapeutic options are.

The slides, text, references are made to help students:

- Know about and understand the strands, aims and stages of sexual development with an understanding of the general developmental steps
- Know about the developmental stages during adolescence and the types and frequency of sexual problems in this life phase
- Know about the impact of pregnancy and of the menopausal transition on sexual function and the hormonal changes in estrogen and testosterone possibly associated with sexual dysfunction
- Know about the impact of aging on sexual function of women and men and the contributing biological factors
- Know and understand the common etiopathogenetic factors during all life phases and based on this the common therapeutic approaches
- Know the algorithm (model of understanding) leading to the individual sexual problems and dysfunctions in the context of diseases and treatment including the history of preexisting difficulties and resources to the disease and treatment specific factors and from there to the response of the patient and the couple confronted with the disease
- Know about the diagnostic process itself applying communicative skills to encourage patients to talk about sexuality and come to a descriptive diagnosis and from there by exploring disease related factors and psychosocial factors arrive at a comprehensive biopsychosocial diagnosis which is the basis for counseling, information, education and shared decision making regarding the individual therapeutic options
- Understand the way from sexual history to treatment in the case of a patient suffering from a cardiovascular disease

***Module 3 is a summarized and shortened form of topic 7 with elements of topic 1, 2, 3.***

Topic 7 Diseases and their impact on Sexuality

- Know about the different factors contributing to the development of sexual dysfunctions in the context of a disease
- Know about the most frequent pre-existing risks and resources
- Know about the main pathways through which a disease and the treatment can impact sexual health
- Know about the stressors and resources for individuals and couples confronted with a disease and their possible impact on sexual health
- Know about the challenges for partners of patients and the relationship in the context of disease and treatment
- Know about the general principles of Sexual Health Care in the context of disease and treatment
- Know about medical and psychosocial factors involved in the development of sexual health symptoms
- Know about the diagnostic approach in medical sexology
- Know about the therapeutic options in medical sexology
- Explain the approach in patients with cardiovascular and oncologic diseases

### Module 4: Diversity, Sexual Minorities, Digitalization and New Sexualities

Module 4 provides basic information about the diversity and variety of sexual expression, the members and naming of the LGBTQIA+ community, the challenges they faced and still face as a minority on a medical, political and legal level. Based on these challenges the specific sexual health needs of these minorities are described and the responses described which should be provided by the laws, institutions and special services provided by trained health care professionals (e.g contraception).

This module provides also basic information about digital technologies (Websites, Apps, Serious Games), their impact on sexual health in general and for the individual person by covering three main areas (sexual health promotion, sexual diversity and clinical sexology).

The slides, text, references are made to help students

- Know and understand the large variety of sexual expression with the 5 dimensions of Sexual Identity, Biological Sex, Gender Expression (social dimension), Sexual orientation (mental dimension), Affective orientation-emotional dimension and the various groups summarized under LGBTQIA+ annotation with overlapping individual expressions
- Understand what is meant by sexual and gender minorities and get an idea about the prevalence of these groups
- Know about the differences in the medical, political and legal environment across time and the globe
- Know about the general and specific health risks for MSM and WSW and the special needs these communities have with respect to Medical Follow up, Psychosocial and Mental Health Care, STI care and care for Sexual Violence Survivors, and Contraception
- Know about the main digital technological tools (Websites, Apps, Serious Games) and the fields and domains in which these tools are used (Sexual Health promotion, Sexual Diversity, Clinical Sexology)
- Be able to give examples of Apps, Games and Websites aiming to increase knowledge and competence in the context of sexual violence, sexual diversity and mental health issues for sexual minorities
- Know about the possibilities for individuals with sexual problems to get online information and partially online treatment in general and know about studies showing efficacy and help for patients with erectile dysfunction, vulvodynia, individuals having committed sexual offense against children
- Know about the possibilities for individual suffering from chronic diseases to get Internet based support and know about studies testing efficacy and satisfaction of user
- Know about the issues and difficulties in implementation of these tools

### Module 5: Compulsive sexual behavior, Paraphilias

This module provides basic information on Compulsive Sexual Behavior (CSB), its definition and diagnostic presentation. It provides the clinical base to assess compulsive sexual behavior disorder according to the ICD11, while also covering alternative formulations, models of understanding and basic interventions. This chapter concludes by providing the differential diagnosis for CSB.

This module also provides basic information regarding Paraphilias and Paraphilic Disorders. Starting from the definitions provided by the DSM5 and ICD11, it covers prevalence and behavioral spectrum; criteria and typology are then presented with each specific diagnostic, concluding with an evidence-based treatment option.

The slides, text, references are made to help students

- Understand CSB definition, prevalence and diagnostic criteria according to available diagnostic tools and different populations
- Learn about the history of CSB and understand the complex clinical presentation, which has elements of overlap with other disorders
- Know about the models of understanding of CSB(D) and of the possible self-reinforcing cycles that maintain this behavior and that justify the variety of expressions that it may assume according to different Authors
- Understand the possible comorbidities and in particular learn how to differentiate between CSB(D) and paraphilic disorders
- Learn about available therapeutic interventions and treatments for CSB(D)
- Understand that CSB(D) as a Disorder is still under research and further research is still needed in this field
- Understand Paraphilias and Paraphilic Disorder definitions, prevalence and diagnostic criteria according to DSM5 and ICD11. Learn about the behavioral spectrum of paraphilias
- Understand the typology presented in the ICD11 and the DSM5, and each specific diagnosis for each typology: Exhibitionism, Voyeurism, Pedophilia, Coercive sexual sadism, Frotteurism and other paraphilic disorders
- Learn about evidence-based treatment options and the level of treatment based on the intensity, compliance and risk of reoffending of the individual paraphilic disorder

### Teaching attitudes and skills

***Video Sexual History Taking***

The videos are based on international guidelines and follow the principles of respect, nonjudgmental attitude, patient centeredness with active listening, mirroring, responding to emotions and summarizing. The communication should take into account that an individual’s sexuality is composed of sexual identity, emotional and sexual orientation, gender identity which develop over the life course. Therefore, sexual history taking should encourage the patient to talk about all these aspects in an environment of trust and respect. The video should be viewed together with students, allow feedback and questions from the student.

The moderator should encourage the students to look at specific elements of the communication:

- The nonverbal communication, the body language etc.
- Which questions are asked and in which way?
- What were the answers?
- Did and if yes when did the patient feel uncomfortable?
- Can students summarize the information describing the sexual identity, sexual health and sexual problems?

***Video sexual counseling***

The video should be viewed together with students. Samples of feedback through questions from the student are:

*Look at the nonverbal communication, the body language etc. Which questions are asked in which way? What were the answers? Did the patient feel uncomfortable and if yes when? How did the counselor structure the conversation? Were there difficult parts? Criticisms etc.?*

***Case discussions***

Various cases related to the modules and topics are presented and discussed (Group discussions).

## Summary

Based on the support of the COST-Action ESMN experts from different countries convened to define the necessary elements and components of a competence-based curriculum for undergraduate university students in sexual health/sexual medicine based on reviews and expert discussions. After having agreed on competencies and learning objectives the content of the knowledge, skills and attitude elements of the program were developed following a modified Delphi procedure of multidisciplinary and multinational collaboration realized in personal and virtual meetings.

Ten topics were developed. Each topic consists of a slide set whereby each slide is accompanied by learning objectives, text, references and questions for self-evaluation. These ten topics build the background library which can be used by the respective teachers. Following the requirements of the ECTS the material of the ten topics was integrated into five modules covering the major themes (sexual health and rights, biology and psychology, threats to sexual health, sexual dysfunctions, life course approach to sexuality including medical sexology, diversity and digitalization, compulsive sexual behavior and paraphilias). For skills training two teaching videos (sexual history taking, sexual counselling) were produced.

The material of the curriculum can be used at different times and in different combinations during the undergraduate curriculum of medicine and psychology.
