# A model curriculum in sexual medicine for undergraduate education in Europe

**DOI:** 10.12688/openreseurope.16146.2

**Published:** 2024-07-29

**Authors:** Carlo Matteo Di Dionisio, Johannes Bitzer, Marianne Greil-Soyka

**Affiliations:** 1 Endocrinology and Medical Sexology (ENDOSEX), Department of Systems Medicine, Universita degli Studi di Roma Tor Vergata, Rome, Lazio, Italy; 2 University Hospital - Department of Obstetrics and Gynecology, Universitat Basel, Basel, Basel-Stadt, Switzerland; 3 Austrian Academy for Sexual Medicine (OEASM), Salzburg, Austria, Austria

**Keywords:** medical education, undergraduate curriculum, sexual health, sexual medicine

## Abstract

Sexual health has been recognized as an essential component of the overall health and wellbeing. The current article aims, first, to review the current state of sexual health education in undergraduate medical curricula, identifying gaps, needs and challenges. The main part of this paper describes the development and content of an undergraduate sexual medicine curriculum based on a clear concept of the competencies students should learn regarding knowledge, skills and attitudes.

The content is based on a biopsychosocial understanding of human sexuality elaborated by international experts from different European countries integrating basic knowledge in biology, psychology, sociocultural and political sciences, preventive medicine, and the various therapeutic approaches to help women, men and couples with sexual health problems on a primary care level. In order to enable students to learn the basic skills of sexual history taking and sexual basic counselling two educational videos were produced.

The material presented is part of the European Collaboration in Science and Technology (COST) supported project European Sexual Medicine Network (ESMN). The material provided can serve universities to give the training as a 25-30 hours course equivalent to 1 ECTS.

## Introduction

The present article aims to describe the process that led to the development of a novel sexual medicine curriculum for undergraduate students of Medicine and Psychology. This curriculum was created throughout 4 years by a working group of international experts under the network association nominated European Sexual Medicine Network (ESMN) ^1^, which is an European Cooperation in Science and Technology (COST) Action (CA18124).

The working group had the following tasks

1) Assess gaps and inequalities regarding undergraduate university education in sexual health, sexual health care and sexual medicine in Europe
2) Analyze the difficulties and challenges regarding the creation of a broadly applicable and suitable program for faculties in different countries
3) Define a competency- based curriculum with learning objectives regarding knowledge, skills and attitudes based on the biopsychosocial model of sexuality and sexual health
4) Elaborate and provide the educational material for use in teaching programs integrated into the existing medical and psychology curriculum

As discussed in section 3 and 4, the rationale at the foundation of this effort lies in the current lack of effective and inclusive sexual health education in undergraduate, first level educational curricula of European universities. The discussion builds up on the current fragmented state of education and the resulting lack of expertise for healthcare professionals in addressing sexual health issues with patients. It follows with an overview of the challenges that may hinder the development and implementation of a comprehensive sexual medicine curriculum.

To fill these gaps and harmonize basic university training in sexual health and sexual medicine, the COST Action ESMN established a working group to elaborate a model curriculum in sexual medicine for undergraduate education in Europe. This working group consisted of experts representing different disciplines and fields involved in sexual medicine and sexual health care and different regions in Europe and beyond (see Acknowledgments).

The results of this shared effort are described in the main part of the final section of this article.

## Methods

For task 1 and 2 an extensive search of all materials related to the topic was carried out in the PubMed and Google Scholar search engines. Relevant research articles focusing on sexual health education in undergraduate medical education published in the period 2006–2023 were included in the review. Relevant articles were selected based upon relevance with the current review objectives and analyzed. Keywords used in the search included sexual health, medical education and curriculum (namely sexual health AND medical education; sexual health AND undergraduate medical education; sexual health AND curriculum). The articles published only in the English language were included for the current review. The collected information is presented in the following two sections.

For tasks 3 and 4 the working group consisting of sexual medicine experts in the field of gynecology, urology, social science, public health, preventive medicine, internal medicine and endocrinology from different European countries had in person and virtual meetings which followed a modified Delphi procedure to achieve consensus ^2^.

For all steps described below the chair and vice chair summarized discussions and inputs coming from the experts, sent the material back to the group members to get feedback and corrections which were then integrated after evaluation. Where necessary this procedure was repeated.

In a first step the group had to define the competencies undergraduates should obtain and the related learning objectives. In the next step the group members were invited to suggest topics to be covered by the program which were considered to be essential parts of basic knowledge. After having agreed on the topics experts were invited to contribute to the topics chosen by sending slides to the chair and vice chair. Each slide had to be accompanied by learning objectives, text, references and self-assessment questions for the students. The chair and vice chair integrated the material into a first format of the 10 topics and sent the proposals out for review and comments from the group. This procedure was repeated several times to arrive at the final format which is shown in the text below and accessible at ESMN website. 

## Results

### Gaps and inequalities regarding education and training in sexual health care across Europe

Sexual health is closely linked to both physical and mental wellbeing ^3– 6^. It encompasses the life of the individual as a fundamental component, constantly evolving and changing with it. Accordingly, sexuality is closely related to general health and for this reason, various medical conditions and treatments can have a negative impact on a person's sexual function, interpersonal relationships and well-being ^7, 8^. The importance to not overlook sexuality appears evident at the time of a cancer treatment, for example, where most often the side effects impact heavily on the interpersonal (intimate) relationship of the patient and may affect therapy adherence ^9^; or, in those cases in which a sexual dysfunction can represent a marker of an underlying, non-diagnosed, illness ^10, 11^. Therefore, it is crucial for healthcare providers to consider and address their patients' sexual health alongside other medical issues.

Various barriers have been identified at the time of a clinical visit, that prevent both healthcare providers and patients to talk freely about sexual health issues. Among these, some of the most frequently reported are: lack of training in communication, fear of offending the patient, discomfort with discussing sexual health, lack of education and competence in treating sexual health issues and lack of time in the appointment ^12^. Patients view sexual health as an important aspect of their well-being and happiness, but they are afraid and may feel ashamed to address intimate issues. They expect their physicians to first open the discussion about sexual health problems ^13– 16^. However, many doctors feel they lack adequate education on sexual health and, consequently, do not routinely inquire about sexuality because they feel incompetent or are afraid to lose too much time ^17– 19^.

A significant contributing factor to this, identified repeatedly in the literature, is the insufficient attention given to sexual health education in medical school ^5, 20, 21^. Despite the broadly acknowledged importance of sexual health education in medicine, many medical schools still do not offer sufficient training on this topic ^19, 22, 23^. This gap in education must be addressed to better equip future physicians with the knowledge, skills and attitudes necessary to provide comprehensive care for their patients ^24^. Medical schools should prioritize the inclusion of sexual health education in their curriculums, providing students with the resources and opportunities to develop their competence in this area. This matters also for those universities that may already implement some components of sexual medicine but do so only for topics related to sexually transmitted diseases, pregnancy, reproductive anatomy, and infertility. A comprehensive sexual medicine education should be composed of a multidisciplinary curriculum, providing a health-oriented, biopsychosocial view of human sexuality ^25, 26^.

Multiple surveys have identified this lack in a variety of countries, especially in the USA, Brazil and Northern Europe ^5, 12, 27, 28^. The European region in particular demonstrates a lack of standardized and unified sexual education inside the existing training curricula ^29, 30^. Results from a survey conducted by the European Federation of Sexology (EFS) ^31^ on existing programs and certifications on sexology in Europe, identified 6 different training models in 25 countries:

- Medical model (in France, Czech Republic, Russia, Poland, Romania and Latvia);
- Clinical model, integrating medical and psychological approaches (in Italy, Netherlands and Turkey);
- distinct training in clinical sexology and human sexuality (in Germany, Austria and Spain);
- Sex therapy model (in Portugal, Austria, Greece, Israel, Croatia, and United Kingdom);
- Human sexuality model (in Belgium and Switzerland);
- Nordic human sexuality model (in Sweden, Denmark, Finland, Norway, Iceland and Estonia).

The available education in the field of sexology typically consists of national programs that encompass a range of courses. These programs may lead to a degree, certificate, or diploma in different areas and levels of sexology *(trained in; doctor in; sexologist; sex therapist; etc.).* The majority of this training focuses on providing additional education to professionals who have already acquired a primary educational level. For this reason, it is more common to find sexual health and medicine education in complementary, master or postgraduate courses.

In the last two decades the postgraduate education in sexual health, sexual medicine and sexology has seen an enormous growth, mainly through European and International Scientific societies. In particular: International Society for Sexual Medicine (ISSM), European Society for Sexual Medicine (ESSM), International society for the Study of Women’s Sexual Health (ISSWSH) and several national societies as well as various universities. The concern remains that sexuality and sexual health care are not well integrated into the undergraduate education for future health care professionals including training in medicine and psychology, as at best sexuality education may be offered as elective courses within the basic health professional curriculum. In most European universities, sexology is not an essential component of the teaching curriculum, even for medical students. This marginalization within the existing educational system makes it difficult to enable future health care professionals to provide basic sexual health care.

### The challenge(s) to create a training program in basic sexual health care for undergraduates

Developing a comprehensive sexual medicine curriculum does not concern only the ability to resume and integrate a variety of multidisciplinary concepts around sexual health. The number of challenges that must be considered is not insignificant as they encompass the whole educational environment of sexual medicine.

The first challenge is to create a curriculum that respects quality criteria, the most important one being that the information provided is **scientifically accurate**, **complete, up-to-date and adapted to its final receivers**. It should not be limited to anatomy and pathology, and should instead provide knowledge about aspects of biology, psychology, and social factors altogether (i.e., the biopsychosocial model of human sexuality) ^32^. It should not overlap with the same depth topics that are already taught in medical courses, but should identify them accordingly, so that students can learn and integrate available knowledge inside a more holistic approach. The development of such an educational package should be guided by inputs from different healthcare specialties and disciplines: obstetrics, gynecology, urology, psychiatry; sex therapists, psychologists, and sexologists; and public health experts ^24, 33^. Another step towards this holistic approach would aim to create a working group composed of international experts from different countries and regions, to assure the inclusion of different sociocultural and political backgrounds important to understand patients’ concerns and sexual problems.

From a didactic point of view the challenge lies in harmonizing topics of human biology, psychology and social and political aspects, without overweighting too much a single topic. The educational level should be comprehensive, but not go beyond the level of the current courses on human anatomy, pathology, etc. It should result in a balanced biopsychosocial educational package on sexual health without deepening or discounting too much. Most importantly, the sexual medicine curriculum should include topics that are missing or not covered enough from the current available courses (i.e. gender minorities and inclusive care, digital sexuality, non-normative sexual behavior, sexuality and aging, sexual coercion and violence, sexual rights, to cite a few) ^24^, and should be aimed at developing those skills that practicing physicians identify as challenging (i.e. sexual history taking) ^26^.

Another challenging aspect is that this curriculum should, where possible, be applied to a larger pool of students other than just those of medical schools. The rationale is simple: given the strict linkage between health and sexuality, it is highly likely that healthcare professionals of all kinds will come across patients who have questions or difficulties related to their sexual health ^24^. Moreover, it is strongly suggested that this should start during the first years of University, rather than the latest and or during residencies, as difficulty in discussing the sexual health of patients emerges prior to graduation ^34^ and is easier to overcome if students can practice during their routine care ^28^.

Effective delivery of the Curriculum to students faces three main challenges.

The first consists of tailoring the program to the social climate of the students. Current generations, studying today at university level, were born and raised through a digital era, to which their ability to learn is connected ^25^. In this sense, implementation through internet platforms, modular and accessible anytime, anywhere to learn and practice at their will could represent a dissemination modality to be preferred, especially considering the evolution of digital technologies for self-help and sexual health ^35^.

The second, far more important, is that this tailored and timed education should connect knowledge acquisition with attitude formation and skills practice. To detach from a purely theoretical approach and instead opt for an experiential learning approach: through the use of roleplay, workshops, case discussion and standardized patients; through (multidisciplinary) team-work and direct constructive feedback from peers and educators. The resulting increase in knowledge and *savoir-faire* allows one to develop a professional attitude, as discussed in the next section.

The third challenging aspect relates to both content creation and student education. A key component of the curriculum is to develop and foster a professional stance. By teaching about the diversity of sexual expression, the curriculum shall prepare to meet patients with different sexual values and expression in respect to one’s own. There is no interest in altering the student’s subjective view of sexuality, but rather the aim is to raise awareness and the ability to maintain an objective, unbiased, non-judgmental evidence-based stance during patient care for a sexual concern ^5, 26^.

Finally, the last challenges regard the mode of implementation, the university structure (faculty, educational board) and the sustainability of the curriculum.

An implementation modality to bring sexual health care in medical schools would be to integrate its components into multiple courses throughout the years of training ^5, 26, 36^. Considering the current limited space of medical schools curricula for new courses, and the general burden placed on students, the above mentioned proposal is not feasible. A solution to this is represented by shorter implementations, such as modules as short as two days of activities, that connect evidence-based knowledge and skills practice. In the literature, this kind of implementation has been preferred by students, implemented by universities ^37^, and has demonstrated positive lasting effects even after ten years ^20^.

Another limitation is represented by a lack of resources, training and time by the faculty of the interested medical school. In this case, external experts could be invited to provide education on sexual health topics, both to students and to willing faculty members ^25^. At the same time, formal approval by the university educational board might become another complexity.

A final challenge concerns the educational curriculum maintenance and follow up. A comprehensive sexual medicine curriculum, as stated, would need to be updated according to new relevant research, alongside the faculty of experts that may present it; it would need an administrative body, to regulate and manage financial and human resources, and a disseminative body, to bring the curriculum to the attention of willing stakeholders and institutions.

Resuming, both students, specialists, researchers and experts have highlighted the need for further sexual medicine education within the undergraduate years. For this reason, a well-timed and implemented curriculum might allow for a seeding process for sexual healthcare, as even a short training program (two days or less) can have a long-lasting impact on the medical competence and confidence in addressing sexual health issues, all to the benefit of patients.

### Competencies and learning objectives of the Curriculum

The appointed working group designed the curriculum based on the concept of a competence-based program integrating knowledge, skills and attitudes ^38^.

Following a thorough analysis, the working group decided that the curriculum should cover ten major topics, providing the necessary background **knowledge** undergraduate student should have with respect to sexual health and basic sexual health care:

Topic 1: Sexual health and sexual rights

- Sexual health concepts, definitions;
- what is sexual health;
- importance of sexual health for general health;
- sexual rights, including the right to sexual pleasure;
- dimensions of sexuality (reproduction, bonding, pleasure, etc.);
- the pleasure based approach linked to wellbeing;
- variety of sexual expression.

Topic 2: Biology of human sexuality

- From genes to bodies;
- biological (anatomical and physiological) processes and constituents of human sexuality;
- development, human response, anatomy, physiology, endocrinology, biological systems approach.

Topic 3: Psychology of human sexuality

- Psychological perspectives, theories, and approaches;
- psychotherapeutic methods to manage with sexual problems;
- barriers and facilitators to lead a self-determined sexual life.

Topic 4: Threats to sexual health, sexuality related health risks; prevention and public health aspects

- Unintended pregnancies and unsafe abortion;
- STIs inc. HIV;
- worldwide prevalences;
- sexual violence;
- prevention, maintenance, and promotion of sexual health.

Topic 5: Female sexual dysfunction

- Definitions and prevalence;
- diagnosis (biopsychosocial);
- types of treatment (biopsychosocial);
- sexual dysfunction and the partner(s).

Topic 6: Male sexual dysfunction

- Definitions and prevalence;
- diagnosis (biopsychosocial);
- types of treatment (biopsychosocial);
- sexual dysfunction and the partner(s).

Topic 7: Medical conditions and sexuality

- Medical illnesses and treatment impact on sexuality;
- pharmacology use and implications (side effects);
- medical conditions (including psychiatric morbidity) and sexuality;
- disability, chronic conditions and sexuality;
- the biopsychosocial approach in medical sexology.

Topic 8: Sexual minorities

- Sexual health of minorities;
- LGBTQI*, sexual needs;
- needs-based care.

Topic 9: Digitalization

- websites, apps, serious games;
- sexual health promotion;
- sexual diversity.

Topic 10: Compulsive sexual behavior; Paraphilias

- Definitions;
- contributing factors and risks;
- legal framework;
- therapeutic approaches.

This knowledge should be transmitted to students through a series of lectures. Given the amount of material, the curriculum can also be organized in a shortened version. This can be accomplished summarizing the knowledge part of the curriculum along 5 modules, of which an example is provided in the next paragraph:

- module 1: sexuality, biology, psychology, sexual medicine;
- module 2: threats to sexual health, sexual dysfunctions;
- module 3: sexuality during the life course, diseases and sexuality;
- module 4: diversity, sexual minorities, digitalization;
- module 5: compulsive sexual behavior, paraphilias.

The **skills** training part of the curriculum should focus on the specific challenges regarding communication and counseling on sexual and reproductive health. It is important to address sexual health issues in a patient centered way and when addressing issues in a couple to respectfully consider both partners perspectives.

Patient centered communication in Sexual Medicine can include a whole range of topics such as reassuring patients of privacy and explaining confidentially, making efforts to ensure trust and openness, and avoiding assumptions (on topics like sexual orientation, gender identity, monogamy, sexual activities, or age-related practices).

This part should be taught by video roleplays and following discussions with students, in particular:

- Video 1: *Sexual history taking based on the concept of the variety of sexual expression*
- Video 2: *How to help patients presenting a sexual problem in the consultation*

During the educational activity, undergraduate students should have space to reflect on their beliefs, values and discomforts regarding sexuality through self-reflection exercises. By taking into account their personal history in relation to sexual health, participants might gain insight on how their individual experience might impact the care they provide. Being more aware and informed, they would be able to implement an open, non-judgmental **attitude**.

Having participated in the course, undergraduate students should be able to:

- Perform a sexual history, including assessment of sexual health risks and sexual concerns, encourage questions and create an open, confidential and non-judgmental atmosphere following the principles of patient centered communication and patient/professional relationship based on trust and respect
- Counsel patients on sexual health protection and promotion (contraception, STIs, violence)
- Encourage questions and inform and educate patients and their partners about the basic facts of the anatomy, physiology and psychology of the human sexual response in women and men and their partners
- Address proactively sexual wellbeing and sexual function in a respectful non-invasive manner
- Take care of patients with sexual problems and concerns in general as well as in the context of disease
  - by practicing patient-centered counseling (including, in case, partner/s) based on proactive respectful asking, empathic listening and providing evidence-based information with respect to the problem presented;
  - by informing patients (and partner/s) about therapeutic options and provide referral if desired/needed;
  - communicate, educate and counsel non only the individual patient but have the skills to include the partner(s) in the process (dyadic setting).

### The final version of the curriculum

After having reached agreement on the main components of the competence-based program in sexual medicine, the working group developed the material to allow universities to implement or integrate the teaching of basic sexual medicine and sexual health-care in existing undergraduate curricula.

 The material can be used according to the needs and curricula of the educational program of universities of medicine and psychology, defined by the respective responsible academic board or dean. Singular topics and/or combination of topics can be integrated into the curriculum in the Bachelor or Master program (divided or total). In universities not following the Bologna system the material can be used according to the respective educational needs.

**Figure 1.**
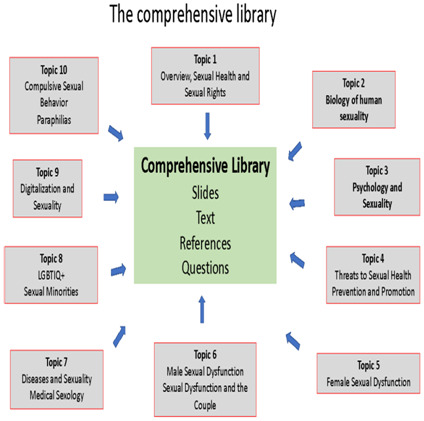
The comprehensive library.

The final version of the curriculum has been developed based on the broad understanding of human sexuality as an experience and behavior which integrates biological and psychosocial factors and processes interacting with each other. Based on this concept the topics agreed upon are covered each by a respective slide set. Each one has defined learning objectives, text, references and questions for self-evaluation. Together, these 10 topics represent the comprehensive library (see Figure 1).

**Figure 2.**
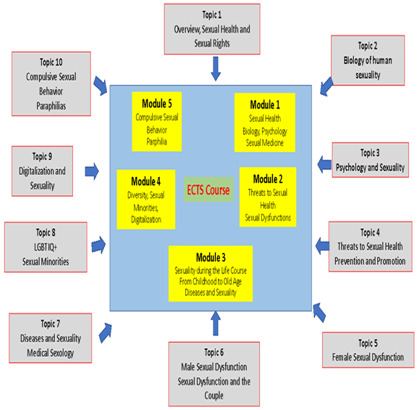
Modules Example.

These topics provide the core body of information which can be used by lecturers, trainers and teachers according to their needs and their special interests. For teaching purposes and to provide an adaptable solution to different curricula’ needs, this core body of information can be structured along modules. In the next paragraph, an example of a 5-module configuration is provided with a general description (see Figure 2).

### Module 1: Overview about definitions of sexual health; basic biology and psychology of human sexuality

Module 1 provides basic knowledge about: definitions of sexual health with the implications for public health programs; multidisciplinary sexual health care for the individual provided by different health care professionals; the main principles and elements of the biology and psychology of the human sexual response.

The slides, text, references are made to help students:

- Describe human sexuality as an integral part of life and behavior and correctly define the concepts of sexual health and sexual rights;
- explain the determinants and contributing factors of sexual health on the international, national and individual level and describe the linkage between sexual health promotion and reproductive health;
- summarize the basic models of understanding of the human sexual response;
- explain the biology of the sexual response from a systemic perspective with the different interactive elements, with top down and bottom up signaling pathways acting together in accordance with the dual control model (exciting, neutral, inhibiting) including the response of the peripheral sexual organs;
- restate the different psychological perspectives and concepts regarding human sexuality from evolutionary perspective to the cognitive behavioral, psychodynamic and systemic approaches;
- list the psychological factors (motivations, thoughts, emotions, behavior, communication) contributing to a self-determined and enjoyable sexuality and the psychological factors hindering and inhibiting the development and realization of this self-expression;
- explain the dual control model of sexual expression from a psychological perspective;
- summarize the key determinants for a pleasure-based approach to sexual health, according to the world association of sexology;
- restate which are the most common reasons for individuals to look for counselling and the wishes and needs of people regarding their sexual health;
- define, based on the biological and psychological factors contributing to the sexual health of the individual, the so-called multidisciplinary approach.

Module 1 is a composition and shortened form of the material produced in topic 1, topic 2 and topic 3. Each topic learning objectives and material is available on the ESMN website. 

### Module 2: Threats to sexual health; sexual dysfunctions

This module provides the basic knowledge about the threats to sexual health as well as the prevention and promotion of sexual health on a social and an individual level and the most frequent sexual dysfunctions in women, men and couples. The diagnostic approach to describe, differentiate and understand these dysfunctions from a biopsychosocial perspective and the various treatment options including medical and psychosocial interventions are demonstrated as overviews.

The slides, text, references are made to help students:

- describe the main threats to sexual health and their impact on physical and mental health in general and on sexual health more specifically;
- summarize the data about the global epidemiology of unintended pregnancies, unsafe abortion, STIs, sexual violence, sexual discrimination and their specific health consequences and implications on sexual health;
- explain the multilevel strategies and instruments for sexual health prevention and promotion (laws and politics, education, sociocultural environment, economy);
- restate the different categories of female and male sexual dysfunctions
- define the biopsychosocial model of sexual function and dysfunction with the four dimensions;
- list the different steps to establish a diagnosis in patients complaining about sexual problems: from listening and history taking to a descriptive diagnosis and from there the comprehensive explanatory diagnosis summarizing the biomedical, psychological and sociocultural factors contributing to the problem. Some of these factors are distant and predisposing, some are precipitating, and some are recent and maintaining the problem;
- summarize the main factors contributing to female and male desire and arousal disorders; to female and male orgasmic disorders; to female and male pain disorder
- define the multidimensional therapeutic approach to help individuals with suffering from sexual dysfunction (basic counselling, medical, psychological, psychosexual interventions, physiotherapy) with examples;
- Identify possible treatment options, paying close attention to treatment impact and possible side effects, especially for pharmacology and specific invasive interventions.

Module 2 is a composition and shortened form of the material produced in topic 4, topic 5 and topic 6. Each topic learning objectives and material is available on the ESMN website. 

### Module 3: Life course approach to sexual health, medical sexology

This module provides basic knowledge about the development of human sexuality along the life course. This explanation takes into account that sexuality is a not a fixed unchangeable dimension of life but that there are typical developmental aims and strands which expose individuals to life-stage-related sexual difficulties, demanding age sensitive sexual health care from childhood to adolescence, midlife to the elderly individuals and couples. Diseases are part of human life and have an impact on sexual health through biological and psychosocial changes. The module provides an understanding of the different levels through which diseases and treatment can affect sexuality, how a sexual dysfunction in the context of a medical condition can be diagnosed and what the therapeutic options are.

The slides, text, references are made to help students:

- describe the strands, aims and stages of sexual development with an understanding of the general developmental steps;
- explain the developmental stages during adolescence and the types and frequency of sexual problems in this life phase;
- recognize the impact of pregnancy and of the menopausal transition on sexual function and the hormonal changes in estrogen and testosterone possibly associated with sexual dysfunction
- recognize the impact of aging on sexual function of women and men and the contributing biological factors
- restate the common etiopathogenetic factors during all life phases and based on this the common therapeutic approaches
- quote the algorithm (model of understanding) leading to the individual sexual problems and dysfunctions in the context of diseases and treatment, including the history of preexisting difficulties and resources to the disease and treatment specific factors and from there to the response of the patient and the couple confronted with the disease;
- summarize the diagnostic process, matching the right communicative skills to encourage patients to talk about sexuality. List the elements needed for a descriptive diagnosis and a comprehensive biopsychosocial diagnosis;
- describe the process from sexual history to treatment in the case of a patient suffering from a cardiovascular disease.

Module 3 is a summarized and shortened form of topic 7 with elements of topic 1, 2, 3. Each topic learning objectives and material is available on the ESMN website.

### Module 4: Diversity, Sexual Minorities, Digitalization and New Sexualities

Module 4 provides basic information about the diversity and variety of sexual expression, the members and naming of the LGBTQIA+ community, the challenges they faced and still face as a minority on a medical, political and legal level. Based on these challenges the specific sexual health needs of these minorities are described, followed by the ideal solutions provided by laws, institutions and special services and by trained health care professionals.

This module provides also basic information about digital technologies (Websites, Apps, Serious Games), their impact on sexual health by covering three main areas: sexual health promotion, sexual diversity and clinical sexology.

The slides, text, references are made to help students:

- list the large variety of sexual expression with the 5 dimensions of sexual identity, biological sex, gender expression (social dimension), sexual orientation (mental dimension), affective orientation-emotional dimension and the various groups summarized under LGBTQIA+ annotation;
- quote what is meant by sexual and gender minorities and match the prevalence of these groups;
- summarize the differences in the medical, political and legal environment across time and the globe;
- describe the general and specific health risks for men who have sex with men (MSM) and women who have sex with women (WSW) and the special needs these communities have with respect to medical follow up, psychosocial and mental health care, STIs, care for sexual violence survivors, and contraception;
- recognize the main digital technological tools (websites, apps, serious games) and the fields and domains in which these tools are used (sexual health promotion, sexual diversity, clinical sexology);
- identify appropriate examples of apps, games and websites aiming to increase knowledge and competence in the context of sexual violence, sexual diversity and mental health issues for sexual minorities;
- summarize the possibilities for individuals with sexual problems to get online information and partially online treatment in general and know about studies showing efficacy and help for patients with erectile dysfunction, vulvodynia, and individuals having committed sexual offense against children;
- explain the possibilities for individual suffering from chronic diseases to get internet based support and know about studies testing efficacy and satisfaction of user;
- restate the issues and difficulties in implementation of these tools.

Module 4 is a summarized and shortened form of topic 8 and 9 with elements of topic 1, 2, 3. Each topic learning objectives and material is available on the ESMN website. 

### Module 5: Compulsive sexual behavior; Paraphilias

This module provides basic information on Compulsive Sexual Behavior (CSB), its definition and diagnostic presentation. It provides the clinical base to assess compulsive sexual behavior disorder according to the ICD11, while also covering alternative formulations, models of understanding and basic interventions. The chapter concludes by providing the differential diagnosis for CSB.

 It follows with basic information regarding Paraphilias and Paraphilic Disorders. Starting from the definitions provided by the DSM5 and ICD11, it covers prevalence and behavioral spectrum; criteria and typology are then presented with each specific diagnostic, concluding with an evidence-based treatment option.

The slides, text, references are made to help students:

- define CSB, CSBD, its prevalence and diagnostic criteria according to available diagnostic tools and different populations;
- recall the history of CSB and the complex clinical presentation, which has elements of overlap with other disorders;
- summarize the models of understanding of CSB(D) and of the possible self-reinforcing cycles that maintain this behavior and that justify the variety of expressions that it may assume;
- list the possible comorbidities and in particular learn how to differentiate between CSB(D) and paraphilic disorders;
- identify available therapeutic interventions and treatments for CSB(D);
- recall that CSB(D) as a Disorder is still under research and further research is still needed in this field;
- match Paraphilias and Paraphilic Disorder definitions, prevalence and diagnostic criteria according to DSM5 and ICD11. Learn about the behavioral spectrum of paraphilias;
- list the typologies presented in the ICD11 and the DSM5, and each specific diagnosis for each typology: exhibitionism, voyeurism, pedophilia, coercive sexual sadism, frotteurism and other paraphilic disorders;
- identify evidence-based treatment options and the level of treatment based on the intensity, compliance and risk of reoffending of the individual paraphilic disorder.

Module 5 is a summarized and shortened form of topic 10 with elements of topic 1 and 3. Each topic learning objectives and material is available on the ESMN website. 

**Completing the library, the curriculum provides video roleplays of a consultation for a sexual complaint, following the steps from first contact to a comprehensive diagnosis following a throughout sexual history interview.** The two videos include a sexual history taking session and a follow-up focused on sexual counselling.

 These videos are based on international guidelines and follow the principles of respect, nonjudgmental attitude, patient centeredness with active listening, mirroring, responding to emotions and summarizing. The communication must take into account that an individual’s sexuality is composed of sexual identity, emotional and sexual orientation, and gender identity. Therefore, sexual history taking should encourage the patient to talk about all these aspects in an environment of trust and respect. The video should be viewed together with moderators, to focus the attention to specific segments. Feedback and questions from the student are welcomed. In particular, the moderator should encourage to look at specific elements of the communication:

- *the nonverbal communication, the body language, etc.*
- *Which questions are asked and in which way?*
- *What were the answers?*
- *Did the patient feel uncomfortable? When?*
- *Are you able to summarize the information provided for the sexual identity, sexual health and sexual problems?*
- *Etc.*

The use of videos is primarily directed at teaching students key concepts of patient centered communication. However, by focusing on verbal aspects, this activity also introduces students to the subject of technology-mediated consultation in sexual medicine (sometimes called E-health; covered in topic 9 of the curriculum). This format has existed since long but was overshadowed by in-person consultations ^39^. In the latest years, especially after the coronavirus 2019 pandemic (COVID-19), It gained significant traction in clinical practice ^40^. It could be helpful for students to consider this tool in their future sexual health consultations.

Finally, complementary case discussions related to the topics’ material are presented in order to elicit group discussions.

## Summary

Based on the support of the COST-Action ESMN, experts from different countries convened to define the necessary elements and components of a competence-based curriculum for undergraduate university students in sexual health/sexual medicine based on reviews and expert discussions. After having agreed on competencies and learning objectives, the content of the program was developed following a modified Delphi procedure of multidisciplinary and multinational collaboration, realized in personal and virtual meetings.

Ten topics were developed. Each topic consists of a slide set whereby each slide is accompanied by learning objectives, text, references and questions for self-evaluation. These ten topics build the background library which can be used by the respective teachers. Following the requirements of European Credit Transfer and Accumulation System ECTS, the topics’ material was integrated into five modules covering the major themes (sexual health and rights, biology and psychology, threats to sexual health, sexual dysfunctions, life course approach to sexuality including medical sexology, diversity and digitalization, compulsive sexual behavior and paraphilias). For skills training two teaching videos (sexual history taking, sexual counselling) were produced.

The material of the curriculum can be used at different times and in different combinations during the undergraduate curriculum of medicine and psychology. Nonetheless, its contents are not limited to training schools, as all the material developed is openly available online. Students who are interested in the material can access it at any moment and virtually anywhere. Further development could allow for an online-based format that could, in theory, satisfy the educational needs of faculties without having to resort to in person events. This area of opportunity will be explored during the next steps of implementation of the curriculum, as it is something already suggested in the literature ^41^.
