# Supplementary material for: A model curriculum in sexual medicine for undergraduate education in Europe
Source: Open Res Eur. 2024 Jul 29;3:153. Originally published 2023 Sep 19. [Version 2] doi: 10.12688/openreseurope.16146.2 (PMC11420616; doi:10.12688/openreseurope.16146.2)
Supplement: Peer review history

# Reviewer report: Reviewer response for version 2

**DOI:** 10.21956/openreseurope.19759.r44400

**Published:** 2024-09-23

**Authors:** Nicola Döring

**Affiliations:** 1 Technische Universität Ilmenau, Ilmenau, Germany

The manuscript describes the development and content of a model curriculum in sexual medicine for undergraduate education in Europe.

 Overall, the study is relevant and the manuscript is readable.

 However, there is room for improvement in several respects:

 1) To ensure transparency and make the manuscript more informative, the authors should make the "comprehensive library" of slides, texts, references, and questions, as well as the two mentioned videos, available in a public repository (e.g., osf.io) and provide links in the article.

 2) Figure 1 and Figure 2 are of very poor visual quality and do not provide any new information, they are basically duplicates - they can both be deleted as they are not helpful to readers. The figures are also not consistent with the text - module titles are not identical (e.g. for Topic 8).

 3) Practical information is missing: Which medical schools have already implemented the curriculum? Which medical schools are currently planning to implement the curriculum? Do medical schools have to pay license fees to use the material? Etc.

 4) Sustainability is unclear. How will it be ensured that the "comprehensive library" will be regularly updated as, for example, standards of care for transgender individuals or digital sexual health information change?

 5) Cultural contextualization remains unclear: How does the curriculum and "comprehensive library" address national differences, such as the criminalization of sex work in Sweden vs. legalization in Germany, with relevant implications for sexual health care for both sex workers and sex work clients?

 6) Language of instruction: It remains unclear in which languages the "comprehensive library" is and will be available. Many undergraduate programs are taught in local languages rather than in English.

 7) The list of experts in the Acknowledgments section would be much more informative to readers if the countries and affiliations of the experts were included.

 8) The abstract uses only binary language ("women, men") and makes gender diverse people (e.g. intersex, transgender, non-binary) invisible.

 9) The abstract also makes the sexual health care needs of children and adolescents invisible by referring only to adults ("women, men").

 10) The article has several typos (e.g. "curriculums", "competence-    based", "knowl-edge").

 11) "The first consists of tailoring the program to the social climate of the students." Social climate is not the right term. The authors seem to be referring to the level of development of information and communication technology, not to "social climate".

 12) "Most importantly, the sexual medicine curriculum should include topics that are missing or under-covered in currently available courses (i.e., gender minorities and inclusive care, digital sexuality, non-normative sexual behavior, sexuality and aging, sexual coercion and violence, sexual rights, to name a few)". While this is true, the modules do not reflect these principles. For example, the module on minorities does not even mention gender minorities only sexual minorities. Important populations such as sex workers and clients of sex workers are not mentioned.

 13) "STIs including HIV" - all acronyms in the paper should be explained.

 14) "communicate, educate and counsel *non* only the individual patient" -> *not* only

 15) "involve the partner(s) in the process (dyadic setting)". If the target patient's partner*s* are involved, it's not a "dyadic setting"

Is the review written in accessible language?

Yes

Are all factual statements correct and adequately supported by citations?

Partly

Are the conclusions drawn appropriate in the context of the current research literature?

Yes

Is the topic of the review discussed comprehensively in the context of the current literature?

Partly

Reviewer Expertise:

media psychology, sexuality research, communication science, social scientific research methods

I confirm that I have read this submission and believe that I have an appropriate level of expertise to confirm that it is of an acceptable scientific standard, however I have significant reservations, as outlined above.

# Reviewer report: Reviewer response for version 2

**DOI:** 10.21956/openreseurope.19759.r42583

**Published:** 2024-08-30

**Authors:** Carey Bayer

**Affiliations:** 1 Morehouse School of Medicine, Atlanta, USA

Thank you to the team for the thorough revisions and responses, most notably in delineating the knowledge, attitude, and skill components throughout the proposed curriculum. As the curriculum is implemented, it is key to allow learners to practice skills as much as possible given that knowledge does not always translate into clinical practice. As evaluation metrics are implemented as well as feedback from learners going through the curriculum, you may want to consider building in more skill objectives/components throughout the modules in addition to the skill-building you have with the videos and simulations (at this point, all the modules are broken down in terms of the knowledge learners should achieve). Feedback from faculty/clinicians/experts is key for the learners in growing in comfort with sexual history-taking.  As the curriculum and evaluation unfold, consider submitting the modules for publication in places such as MedEdPORTAL, https://www.mededportal.org/ or other accessible platforms for faculty training health professionals.  Great work! I look forward to seeing the outcomes of your hard work in helping health professionals address sexual health with patients across the life course.

Is the review written in accessible language?

Partly

Are all factual statements correct and adequately supported by citations?

Yes

Are the conclusions drawn appropriate in the context of the current research literature?

Partly

Is the topic of the review discussed comprehensively in the context of the current literature?

Yes

Reviewer Expertise:

NA

I confirm that I have read this submission and believe that I have an appropriate level of expertise to confirm that it is of an acceptable scientific standard.

# Reviewer report: Reviewer response for version 1

**DOI:** 10.21956/openreseurope.17430.r36582

**Published:** 2024-01-18

**Authors:** Mark B. Woodland, Priyanka Raju

**Affiliations:** 1 Tower Health, Reading, Pennsylvania, USA; 2 Obstetrics and Gynecology, Reading Hospital, West Reading, Pennsylvania, USA

Inadequate attention is given to sexual health education in medical schools globally. Despite its acknowledged importance, many medical institutions lack comprehensive training on sexual health, resulting in healthcare professionals feeling unequipped to address such concerns. This article suggests that incorporating sexual health education into medical school curriculums is crucial to equip future physicians with the necessary knowledge and skills.

 The authors emphasize the intricate connection between sexual health and well-being, highlighting its significance within an individual's life and appropriately recognize the reluctance of healthcare providers and patients to openly discuss sexual health during clinical interactions due to barriers including  fear, discomfort, and time constraints not to mention lack of training for clinicians.  .

 Obstacles in training have included the lack of a standardized curriculum of expectations for the learners as well as the medical educators.  The authors summarize the existing models of sexual health education across different European countries, showcasing varying approaches and degrees of incorporation within national programs emphasizing the need to integrate sexual health education into undergraduate studies for healthcare professionals.

 In our opinion, the challenges in designing a comprehensive sexual medicine curriculum for undergraduates are reviewed accurately and concisely. These challenges encompass ensuring a scientific, biopsychosocial approach, incorporating diverse topics, adopting effective delivery methods, aligning with students' learning styles in the digital age, and overcoming institutional constraints to facilitate educators in providing the necessary education. In 2017, the World Health Organization (WHO) attempted to create an operational approach to sexual and reproductive health to create a broader awareness of both while not losing sight of crucial elements of sexual health that are emphasized in this article. The focus of this document includes laws, policies, regulations and strategies in addition to cultural and social norms, gender and socioeconomic inequalities and basic human rights (1).

 The major conclusions of the article are summarized into a proposed curriculum structured around competencies, knowledge, skills, and attitudes that undergraduate students should acquire including ten major topics; sexual health concepts, biology of human sexuality, psychological aspects, sexual dysfunctions, sexual minorities, digitalization, and more. The curriculum aims to equip students with the ability to take sexual histories, counsel patients, and address sexual concerns in a professional, empathetic, and non-judgmental manner. To enhance the coverage of these ten areas,  the key components of the curriculum are delivered in a modular format:

 Module 1 covers fundamental knowledge about sexual health definitions, public health implications, and multidisciplinary care.

 Module 2 outlines threats to sexual health, focusing on prevention, sexual dysfunctions in men, women, and couples, diagnostic approaches, and multidimensional therapeutic interventions.

 Module 3 discusses exploration of sexual development across life stages, understanding diseases' impact on sexual health, diagnostic processes, and therapeutic approaches.

 Module 4 reviews sexual diversity, emphasizing the challenges, special health needs, and responses including digital technologies' impact on sexual health and minority-specific healthcare.

 Module 5 provides specific insights into compulsive sexual behavior (CSB), its diagnosis, models of understanding, interventions, and differentiation from paraphilic disorders.

 The article does describe Video Sexual History Taking and includes educational videos showcasing respectful, patient-centered sexual history taking and counseling. Emphasizes nonverbal cues, questioning techniques, comfort levels, and the summarization of gathered information. However, the concept of telemedicine and sexual health is still developing. A 2011 survey looked at younger people (age 16-24) and their experience using telemedicine in sexual healthcare. Although only 29% of respondents were willing to have a webcam consultation, such a service may benefit youth who may not otherwise access sexual health services (2). Given the movement in telemedicine since the pandemic, this might have to be re-evaluated and included with clinician training. Additionally, simulation to help elucidate issues raised in each module have been shown to be a meaningful adjunct in education and could be utilized with this curriculum (3, 4).

 Overall, the article underlines the need for a standardized, comprehensive, and integrated sexual health education curriculum in medical schools to better prepare future healthcare professionals in addressing patients’ sexual health concerns competently and sensitively. The authors build upon the proposal of others who have proposed the need for formal education in sexual health for healthcare providers by developing a comprehensive curriculum composed of ten topics structured around competencies, knowledge, skills, and attitudes that undergraduate students should acquire (5, 6).

 We congratulate the authors on this comprehensive review of sexual health education curriculum. The authors review existing models and educational curricula in detail. The conclusions provided in the article include: 1) an assessment of gaps and inequalities in undergraduate university education, 2) an analysis of the challenges surrounding creation of sexual health curriculum, 3) development a competency-based curriculum based on the bio psychosocial model of sexual health, and 4) creation of education material for use in undergraduate programs. To the best of our knowledge, all factual statements are correct and adequately supported by the listed citations.

Is the review written in accessible language?

Yes

Are all factual statements correct and adequately supported by citations?

Yes

Are the conclusions drawn appropriate in the context of the current research literature?

Yes

Is the topic of the review discussed comprehensively in the context of the current literature?

Yes

Reviewer Expertise:

Obstetrics, gynecology, sexual health, medical education, diversity, equity, and inclusion in medicine and healthcare

We confirm that we have read this submission and believe that we have an appropriate level of expertise to confirm that it is of an acceptable scientific standard, however we have significant reservations, as outlined above.

## References

[1] : Young people's views on the potential use of telemedicine consultations for sexual health: results of a national survey. *BMC Infect Dis* .2011;11: 10.1186/1471-2334-11-285 285 10.1186/1471-2334-11-285 22026640 PMC3213067

[2] : Telehealth sexual and reproductive health care during the COVID‐19 pandemic. *Medical Journal of Australia* .2021;215(8) : 10.5694/mja2.51219 371-372 10.5694/mja2.51219 PMC844706034374090

[3] : The State of Sexual Health Education in U.S. Medicine. *American Journal of Sexuality Education* .2014;9(1) : 10.1080/15546128.2013.854007 65-80 10.1080/15546128.2013.854007

[4] : Sexual Health Education in Obstetrics and Gynecology (Ob-Gyn) Residencies-A Resident Physician Survey. *J Sex Med* .2021;18(6) : 10.1016/j.jsxm.2021.03.005 1042-1052 10.1016/j.jsxm.2021.03.005 34020923

[5] : Improving Inclusive Communication: Pilot Results from a Simulation-Based Learning Opportunity to Practice Taking a Sexual Health History. *Journal of Maine Medical Center* .2023;5(2) : 10.46804/2641-2225.1157 10.46804/2641-2225.1157

# Response

**Published:** 2024-01-29

**Authors:** Carlo Matteo Di Dionisio

**Affiliations:** Universita degli Studi di Roma La Sapienza, Rome, Lazio, Italy

Dear Dr.Woodland, Dear Dr. Raju  

 We are grateful for your thorough reading of our article and your constructive remarks. We will carefully review each of your suggestions as we make revisions and improvements. Rest assured that we will incorporate your recommendations to complete the article. Thank you for your valuable feedback.

# Reviewer report: Reviewer response for version 1

**DOI:** 10.21956/openreseurope.17430.r35221

**Published:** 2023-10-18

**Authors:** Carey Bayer

**Affiliations:** 1 Morehouse School of Medicine, Atlanta, USA

The authors present a well-detailed discussion of the 4-year process undertaken by a working group of experts with varied expertise in sexual health to develop a sexual health curriculum for European undergraduate medical education. The group acknowledges the complexities in not only the topic, but more importantly the training of the topic throughout medical education. 

 The strengths of the piece include the depth of expertise included, the anchoring to the WHO definition of sexual health, the biopsychosocial-lifespan approach taken, the attempt to delineate knowledge/skills/attitudes needed to provide comprehensive sexual health care, the detailing of the topic/module breakdown, the development of 2 videos to accompany the curriculum, and the acknowledgment that the curriculum may be implemented in various ways depending on the needs of each individual program.

 The areas where the manuscript can continue to be strengthened include:

- Discussion of the role of pleasure in addition to well-being early on in the framing
- Including education professionals as a part of the team to help revise the objectives in a more measurable fashion (consider the work of Bloom's Taxonomy, Kirkpatrick levels of measurement) -- for example one action verb per objective and utilizing appropriate action verbs for knowledge vs. attitude vs. skill objectives, "Identify..."Weigh..." "Demonstrate"
- Consider further delineation in the sections to better detail which are the knowledge objectives vs. attitude objectives vs. skill objectives as this will help with what to implement when and determine which is foundational before moving to next steps (i.e. learning definitions/diagnoses before practicing history-taking)
- Consider building in a section to reflect on the learner's history related to sexual health (identifying culture, values/attitudes/beliefs, discomforts) and how personal experiences may impact care provided
- Consider either shifting the gendered modules to "cisgender women" "cisgender men" or combining them to include "across genders" and include cisgender and transgender populations
- In the discussion of treating, make sure to include a discussion of sexual pharmacology and the impact of medication effects on sexual health
- In the discussion of various populations not often included, make sure to include those with disabilities and chronic conditions
- The framing mentions "couples" many times throughout and given the variety of relationship structures today, the group may want to consider an approach in training that allows learners to be prepared to care for any patient regardless of relationship structure instead of possible implicit expectations of being in a coupled relationship structure
- Consider including evaluation metrics with the curriculum (which will be easier to include with measurable action verbs across the objectives)
- Here are a few additional resources that may be helpful as the work evolves: 
  Sexual Health Competencies for Undergraduate Medical Education in North America ^1^
  While this resource focuses on specific populations, it includes great examples of materials, cases, evaluation metrics:
https://store.aamc.org/implementing-curricular-and-institutional-climate-changes-to-improve-health-care-for-individuals-who-are-lgbt-gender-nonconforming-or-born-with-dsd-a-resource-for-medical-educators.html
  Here are examples of other videos you may find helpful for your curriculum:
https://www.aamc.org/about-us/equity-diversity-inclusion/lgbt-health-resources
- There were a few areas in the manuscript where punctuation was missing/typographical errors; please edit

Thanks for your much needed work in this area!

Is the review written in accessible language?

Partly

Are all factual statements correct and adequately supported by citations?

Yes

Are the conclusions drawn appropriate in the context of the current research literature?

Partly

Is the topic of the review discussed comprehensively in the context of the current literature?

Yes

Reviewer Expertise:

My areas of expertise are sexuality education & training health professionals; curriculum development, implementation, and assessment

I confirm that I have read this submission and believe that I have an appropriate level of expertise to confirm that it is of an acceptable scientific standard, however I have significant reservations, as outlined above.

## References

[1] : Sexual Health Competencies for Undergraduate Medical Education in North America. *J Sex Med* .2017;14(4) : 10.1016/j.jsxm.2017.01.017 535-540 10.1016/j.jsxm.2017.01.017 28202322

# Response

**Published:** 2023-10-23

**Authors:** Carlo Matteo Di Dionisio

**Affiliations:** Universita degli Studi di Roma La Sapienza, Rome, Lazio, Italy

Dear Dr. Bayer, Thank you for your invaluable insights and precise observations. The references suggested will be of great help.

 We would like to address your points as follows:

 - Regarding point 1, 6, and 7, the material provided indeed covers the role of pleasure (Topic 1), the impact of pharmacology on sexuality (across various Topics, such as 5, 6 and 7) and sexuality in the presence of disabilities or chronic conditions (Topic 7). We will review the contents described in the article to be more extensive and include the ones above.  

 - For points 2, 3 and 9, involvement of professionals with a background in education is at the top of our priorities. We will focus on delineating knowledge, attitude, and skills objectives (as suggested) for each subject and introducing specific exercises following each topic. Connected to your point 4, in the drafts currently available one of these comes close to your suggestion.

   - Concerning points 5 and 8, a careful revision of gender specific language, including the dyadic relationship framework, will be conducted. We commit to a thorough review, to ensure more inclusivity and widening nonbinary aspects. 

 - In conclusion, we will review the article to provide a more balanced and polished presentation. We have also noted the missing punctuation and errors within the manuscript, that we will edit and rectify. Your expertise offers us potential to grow as educators, and we look forward to presenting a comprehensive version of the curriculum in the near future.
